# Comparative transcriptomics enlarges the toolkit of known developmental genes in mollusks

**DOI:** 10.1186/s12864-016-3080-9

**Published:** 2016-11-10

**Authors:** A. L. De Oliveira, T. Wollesen, A. Kristof, M. Scherholz, E. Redl, C. Todt, C. Bleidorn, A. Wanninger

**Affiliations:** 1 Department of Integrative Zoology, Faculty of Life Sciences, University of Vienna, Althanstraße 14, Vienna, 1090 Austria; 2 University of Bergen, University Museum, The Natural History Collections, Allégaten 41, 5007 Bergen, Norway; 3 Museo Nacional de Ciencias Naturales, Spanish National Research Council (CSIC), José Gutiérrez Abascal 2, Madrid, 28006 Spain; 4 Institute of Biology, University of Leipzig, Leipzig, 04103 Germany

**Keywords:** Bioinformatics, EvoDevo, Evolution, Evolutionary developmental biology, Genomics, Mollusca, Next-generation sequencing, NGS, Transcriptomics, RNA-seq

## Abstract

**Background:**

Mollusks display a striking morphological disparity, including, among others, worm-like animals (the aplacophorans), snails and slugs, bivalves, and cephalopods. This phenotypic diversity renders them ideal for studies into animal evolution. Despite being one of the most species-rich phyla, molecular and *in silico* studies concerning specific key developmental gene families are still scarce, thus hampering deeper insights into the molecular machinery that governs the development and evolution of the various molluscan class-level taxa.

**Results:**

Next-generation sequencing was used to retrieve transcriptomes of representatives of seven out of the eight recent class-level taxa of mollusks. Similarity searches, phylogenetic inferences, and a detailed manual curation were used to identify and confirm the orthology of numerous molluscan Hox and ParaHox genes, which resulted in a comprehensive catalog that highlights the evolution of these genes in Mollusca and other metazoans. The identification of a specific molluscan motif in the Hox paralog group 5 and a lophotrochozoan ParaHox motif in the *Gsx* gene is described. Functional analyses using KEGG and GO tools enabled a detailed description of key developmental genes expressed in important pathways such as Hedgehog, Wnt, and Notch during development of the respective species. The KEGG analysis revealed *Wnt8*, *Wnt11*, and *Wnt16* as Wnt genes hitherto not reported for mollusks, thereby enlarging the known Wnt complement of the phylum. In addition, novel *Hedgehog* (*Hh*)*-*related genes were identified in the gastropod *Lottia* cf. *kogamogai*, demonstrating a more complex gene content in this species than in other mollusks.

**Conclusions:**

The use of *de novo* transcriptome assembly and well-designed *in silico* protocols proved to be a robust approach for surveying and mining large sequence data in a wide range of non-model mollusks. The data presented herein constitute only a small fraction of the information retrieved from the analysed molluscan transcriptomes, which can be promptly employed in the identification of novel genes and gene families, phylogenetic inferences, and other studies using molecular tools. As such, our study provides an important framework for understanding some of the underlying molecular mechanisms involved in molluscan body plan diversification and hints towards functions of key developmental genes in molluscan morphogenesis.

**Electronic supplementary material:**

The online version of this article (doi:10.1186/s12864-016-3080-9) contains supplementary material, which is available to authorized users.

## Background

Over the past decade, an ever increasing number of molecular data has become available for representatives of numerous animal phyla. It has been shown that many genes are evolutionary conserved, either sharing similar functions or being co-opted into various novel functions, thereby often displaying astounding functional plasticity during animal development (e.g., [1–3]). A substantial body of evidence suggests that evolutionary changes or variations in the regulation of highly conserved developmental genes, as well as divergence in gene sequences (e.g., duplications and mutations), have been responsible for major alterations in the evolution of animal body plans [4–6]. Within these conserved genes, two families of homeotic genes that encode transcription factors and are involved in bilaterian anterior-posterior axis and/or digestive tract patterning, the Hox and ParaHox genes, are among the best-investigated so far [7–9]. Therefore, understanding and reconstructing the evolutionary history of these gene families is crucial for inferring animal evolution and the relationships between genetic and morphological complexity [10, 11].

Comparisons between Hox and ParaHox gene clusters support the hypothesis that both families evolved from an early duplication of an ancient ProtoHox cluster [12–15]. Thereby, the Hox and ParaHox clusters underwent different evolutionary pathways, in which the Hox cluster expanded by several tandem duplications, whereas the ParaHox cluster, composed of *Gsx* (paralog of the anterior Hox genes), *Cdx* (paralog of the *Hox3* gene), and *Xlox* (paralog of the posterior Hox genes), did not. Within Lophotrochozoa, a major group of protostome animals that often show a spiral cleavage pattern and/or a ciliated larva in their life cycle, the Hox and ParaHox families are usually composed of 11 and three genes, respectively [16]. Although the majority of studies are restricted to two lophotrochozoan phyla (Mollusca and Annelida), these results suggest that the last common ancestor of all lophotrochozoan animals also harbored a toolkit that included 11 Hox and three ParaHox genes.

The phylum Mollusca comprises approximately 200,000 living species, ranking it the second-most speciose metazoan phylum [17]. Most mollusks, like numerous other lophotrochozoans, display a highly conserved pattern of spiral cleavage in the early embryo, resulting in the formation of four vegetal macromeres and four animal micromeres. In many basally branching clades, embryology is followed by indirect development via a free-swimming, ciliated trochophore-like larva which most likely constitutes the ancestral condition for Mollusca. This type of larva is commonly found in caudofoveates (= chaetodermomorphs) [18], polyplacophorans [19], gastropods [20], scaphopods [21–23], and bivalves (e.g., [24, 25]; see [26] for review). Many gastropods and bivalves develop a secondary, planktotrophic larva, the veliger, while solenogasters (= neomeniomorphs) and protobranch bivalves have independently evolved a secondary lecithotrophic larval type, the so-called pericalymma or test cell larva (see [26] for review; [27–29]).

In evo-devo research, mollusks occupy an important role in studies focused on the function and expression of regulatory genes during development, providing insights into the mechanisms that underlie the diversification of metazoan body plans [30]. To this end, several transcriptomic studies focusing on biomineralisation processes and their concordant genes have recently become available [31–34]. However, given the high morphological disparity, the complex life cycles, and the striking variation during the ontogeny among molluscan taxa, there is a considerable lack of molecular studies dealing with the expression of key developmental genes in this phylum. As such, only a few gene expression studies have been published, including Hox genes [35–41] and ParaHox genes [42–44]. These studies suggest a high plasticity and recruitment into novel functions of these genes at least in cephalopods and gastropods. Since these data stem from very few species only, the full complement of Hox and ParaHox gene expression domains (and hence their putative functions) in Mollusca is yet to be analysed. To this end, an improvement of the equally poor database of other molluscan developmental genes will significantly contribute to further insights into the molecular toolkit that governs key developmental processes of this important lophotrochozoan phylum [45, 46].

With the advent of next-generation sequencing technologies (e.g., [47, 48]), large-scale comparative genomic surveys of non-model species are now possible, allowing for deeper insights into ancestral versus novel features of the molecular machinery that underlies the ontogenetic establishment of animal body plans. Recently, four important molecular resources were established by sequencing and annotating complete genomes for mollusks using the bivalves *Crassostrea gigas* [49] and *Pinctada fucata* [50], the gastropod *Lottia gigantea* [16], and the cephalopod *Octopus bimaculoides* [51] as model organisms. Apart from useful insights into genome organisation and the structure of individual genes in these species, the studies identified the complete Hox and ParaHox complements, adding valuable knowledge about the diversity of these homeotic genes in mollusks.

To expand this database, we sequenced transcriptomes sampled from distinct developmental stages and provide in-depth analyses of the Hox and ParaHox gene families in representative species of seven out of the eight recent class-level taxa of mollusks. Furthermore, we screened our sequences for orthologs present in the Wnt, Notch, and Hedgehog signaling pathways. These highly conserved pathways contribute to orchestrating the broad display of morphology diversity found in bilaterians through epigenetic interactions between cells and the entrainment of certain developmental programs (for review, see [52]). In addition, we provide a broad functional characterisation of the molluscan gene content using Gene Ontology (GO) terms and Kyoto Encyclopedia of Genes and Genomes (KEGG) pathways.

## Results

### Pre-processing and *de novo* assembly of the transcriptomic libraries

The filtering pipeline discarded between 4.78 % (the bivalve *Nucula tumidula*) and 17.40 % (the neomeniomorph = solenogaster *Wirenia argentea*) of low-quality, adaptor contaminated, paired-end reads from the molluscan libraries (Table 1). The assembling process generated high-quality transcriptomes ranging from 34,794 (the gastropod *Lottia* cf. *kogamogai*) to 394,251 (*W. argentea*) sequences (Table 2). The difference in the number of reconstructed base pairs, transcripts, the values of the largest transcript, and the N50 (median transcript length) are obvious between 454 and Illumina libraries. The best 454 library (the cephalopod *Idiosepius notoides*) includes considerably less transcripts, base pairs, and N50 transcript length than any of the short-read Illumina libraries. To facilitate the downstream analysis, both assembled libraries derived from the cephalopod *I. notoides* were combined.

**Table 1:** Summary of the pre-processing pipeline in the molluscan transcriptomic libraries

Organism	No. of reads^a^ before pre-processing	No. of reads^a^ after pre-processing	No. of reads^a^ excluded
*Gymnomenia pellucida* (Neomeniomorpha)	53,751,440	50,292,634 (93.57 %)	3,458,806 (6.43 %)
*Wirenia argentea* (Neomeniomorpha)	50,456,889	41,678,466 (82.60 %)	8,778,423 (17.4 %)
*Scutopus ventrolineatus* (Chaetodermomorpha)	43,492,046	40,596,155 (93.34 %)	2,895,891 (6.66 %)
*Acanthochitona crinita* (Polyplacophora)	35,737,364	33,695,610 (94.29 %)	2,041,754 (5.71 %)
*Idiosepius notoides* ^*b*^ (Cephalopoda)	588,878	588,878 (100 %)	-
*Idiosepius notoides* (Cephalopoda)	38,267,214	35,131,600 (91.81 %)	3,135,614 (8.19 %)
*Lottia* cf. *kogamogai* ^*b*^ (Gastropoda)	402,814	402,814 (100 %)	-
*Nucula tumidula* (Bivalvia)	40,797,848	38,849,372 (95.22 %)	1,948,476 (4.78 %)
*Antalis entalis* (Scaphopoda)	24,194,021	22,881,795 (94.58 %)	1,312,226 (5.42 %)

^a^Read pairs for Illumina libraries

^b^Note that the 454 datasets were just trimmed and converted to fasta and fasta.qual files. The quality and length filtering was executed by the program MIRA4 during the assembling step

**Table 2:** Summary of assembly statistics from the nine molluscan transcriptomic libraries

Assembler	Organism	No. of transcripts	No. of transcripts > 1,000 bp	No. of reconstructed bases (bp)	No. of reconstructed bases in transcripts >1,000 bp	Length of the largest transcript reconstructed (bp)	N50
IDBA-tran	*Gymnomenia pellucida* (Neomeniomorpha)	228,678	136,889	408,484,174	355,797,467	26,833	2,616
	*Wirenia argentea* (Neomeniomorpha)	394,251	178,721	495,209,150	369,131,155	13,881	1,725
	*Scutopus ventrolineatus* (Chaetodermomorpha)	220,258	96,068	253,037,497	181,977,467	17,067	1,555
	*Acanthochitona crinita* (Polyplacophora)	364,800	234,607	689,247,497	614,059,419	17,023	2,737
	*Idiosepius notoides* (Cephalopoda)	285,863	93,114	297,178,066	189,330,826	19,705	1,399
	*Nucula tumidula* (Bivalvia)	273,272	126,403	378,309,195	296,427,272	20,605	2,100
	*Antalis entalis* (Scaphopoda)	351,943	125,869	369,111,329	241,658,022	28,825	1,399
MIRA4	*Idiosepius notoides* (Cephalopoda)	43,218	6,880	29,267,478	10,095,956	10,063	785
	*Lottia* cf. *kogamogai* (Gastropoda)	34,794	6,391	25,737,707	9,530,625	7,134	817

### Identification of the coding sequence regions and clustering of the transcriptomes

This procedure generated high-quality redundant protein gene sets that contained between 17,163 (*Lottia* cf. *kogamogai*) and 216,221 (the polyplacophoran *Acanthochitona crinita*) sequences. The percentage of transcripts in each of the molluscan libraries that codes for a putative protein sequence ranges from 21 % (*Idiosepius notoides*) to 59 % (*A. crinita*) (Table 3). After the clustering and the elimination of protein sequence redundancy, the number of sequences lowered by more than 70 % in some protein gene sets (approx. 74 % in *Wirenia argentea*, approx. 72 % in *A. crinita*, and approx. 71 % in the scaphopod *Antalis entalis*). The 454 protein gene set derived from *L.* cf. *kogamogai* showed the lowest reduction in the number of protein sequences, in which just more than approx. 2 % of the sequences were clustered.

**Table 3:** Summary of empirical homology-based prediction and clustering methodology in the molluscan transcriptomic libraries

Organism	No. of transcripts	No. of possible putative proteins	No. of selected putative proteins	No. of non-redundant putative proteins
*Gymnomenia pellucida* (Neomeniomorpha)	228,678	834,304	125,766	54,997
*Wirenia argentea* (Neomeniomorpha)	394,251	1,185,594	213,616	54,183
*Scutopus ventrolineatus* (Chaetodermomorpha)	220,258	499,165	87,291	39,631
*Acanthochitona crinita* (Polyplacophora)	364,800	1,663,283	216,221	59,271
*Idiosepius notoides* (Cephalopoda)	329,081	543,405	70,861	21,533
*Lottia* cf. *kogamogai* (Gastropoda)	34,794	47,120	17,163	16,781
*Nucula tumidula* (Bivalvia)	273,272	787,355	105,381	38,563
*Antalis entalis* (Scaphopoda)	351,943	739,709	124,738	35,443

### Assessment of the protein gene set completeness using BUSCO

The completeness in the molluscan protein gene sets, as approximated by the presence of universal single copy orthologs [53], showed an ample variability ranging from 68.21 % (*Lottia* cf. *kogamogai*) to 95.02 % (*Nucula tumidula*) (Table 4). A correlation of completeness and the sequencing technique is noticeable among the different molluscan protein gene sets. For instance, the most incomplete protein gene set using deep Illumina sequencing (the chaetodermomorph = caudofoveate *Scutopus ventrolineatus*: 79.83 % of completeness) is more complete than the one generated by the 454 pyrosequencing (*L.* cf. *kogamogai*: 68.21 % of completeness). Likewise, the number of fragmented BUSCOs in the *S. ventrolineatus* library is still lower than the number of fragmented BUSCOs in the *L*. cf. *kogamogai* 454 sequenced library. The statistics of pre-processing, assembly, and quality assessment pipelines are summarised in Table 5.

**Table 4:** BUSCO summary of the molluscan protein gene sets

Organism	Complete Single-copy BUSCOs	Fragmented BUSCOs	Missing BUSCOs	Completeness (%)
*Gymnomenia pellucida* (Neomeniomorpha)	708	87	48	94.31
*Wirenia argentea* (Neomeniomorpha)	450	234	159	81.14
*Scutopus ventrolineatus* (Chaetodermomorpha)	506	167	170	79.83
*Acanthochitona crinita* (Polyplacophora)	660	136	47	94.42
*Idiosepius notoides* (Cephalopoda)	680	102	61	92.76
*Lottia* cf. *kogamogai* (Gastropoda)	286	289	268	68.21
*Nucula tumidula* (Bivalvia)	705	96	42	95.02
*Antalis entalis* (Scaphopoda)	697	100	46	94.54

**Table 5:** Summary of initial pre-processing and generation of the high-quality molluscan protein gene sets

Organism	No. of raw reads^a^	No. of reconstructed transcripts	No. of n-r^b^ putative proteins	Gene set completeness (%)
*Gymnomenia pellucida* (Neomeniomorpha)	53,751,440	228,678	54,997	94.31
*Wirenia argentea* (Neomeniomorpha)	50,456,889	394,251	54,183	81.14
*Scutopus ventrolineatus* (Chaetodermomorpha)	43,492,046	220,258	39,631	79.83
*Acanthochitona crinita* (Polyplacophora)	35,737,364	364,800	59,271	94.42
*Idiosepius notoides* (Cephalopoda)	38,267,214	285,863	21,533^c^	92.76^c^
*Idiosepius notoides* (454) (Cephalopoda)	588,878	43,218	–	–
*Lottia* cf. *kogamogai* (Gastropoda)	402,814	34,794	16,781	68.21
*Nucula tumidula* (Bivalvia)	40,797,848	273,272	38,563	95.02
*Antalis entalis* (Scaphopoda)	24,194,021	351,943	35,443	94.54

^a^Read pairs for Illumina libraries

^b^Non-redundant

^c^After the assembly step the *Idiosepius notoides* libraries were combined together for the subsequent downstream analysis

### Identification of Hox and ParaHox sequences and phylogenetic analyses

**Fig. 1:**
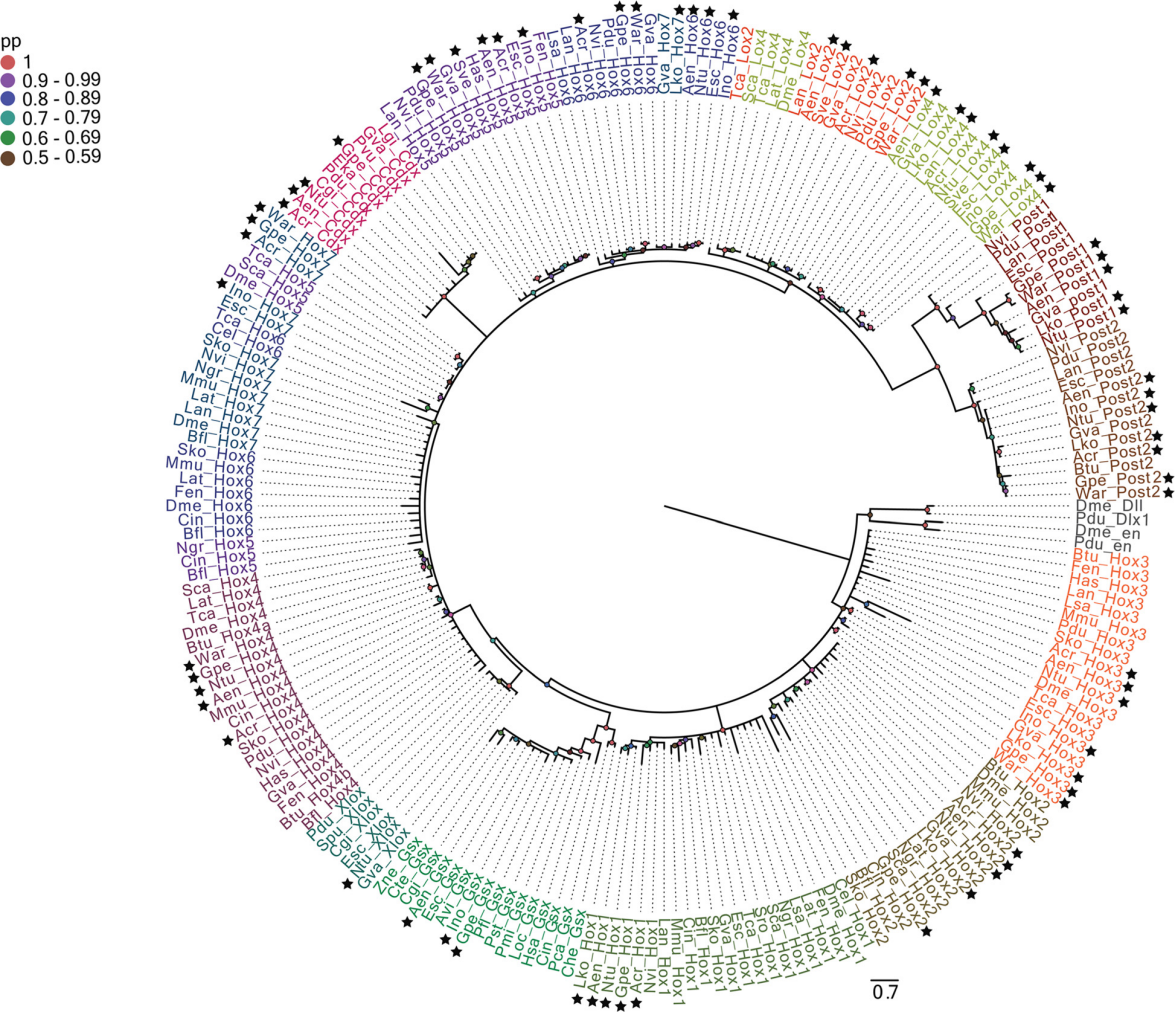
Phylogeny of Hox and ParaHox genes from amino acid sequences containing homeodomain and flanking regions. The consensus tree was inferred by Bayesian phylogenetic analysis with MrBayes v3.2.2 discarding 25 % of the sampled trees as burn-in. The branch support values are posterior probability values. The new Hox and ParaHox sequences identified in this study are highlighted by black stars. Hox and ParaHox paralog groups are represented by different colors. The homeobox genes *distalless* and *engrailed* were used as outgroups

**Fig. 2:**
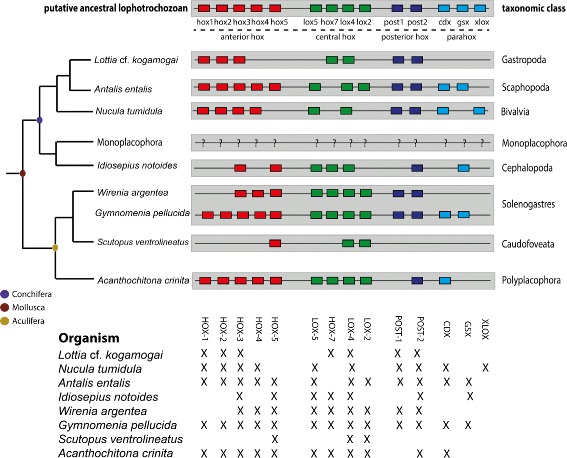
Summary of the Hox and ParaHox genes identified in the eight molluscan species studied herein. For comparison, the putative ancestral lophotrochozoan gene toolkit is provided. Tree topology follows Smith et al. [61]. The colored boxes indicate the anterior, central, and posterior Hox as well as the ParaHox paralog groups. To date, no known Hox or ParaHox sequences (represented by question marks) belonging to a monoplacophoran mollusk have been identified. The colored circles in the branch nodes represent the last common ancestor of the monophyletic clades Conchifera, Aculifera, and Mollusca, respectively. It is important to note that the figure does not depict the chromosomal organisation of the Hox and ParaHox genes in the studied species. Thus, it is well possible (and in case of the cephalopod *Idiosepius* even likely) that the Hox complement is not organised in a distinct cluster in (some of) the species depicted herein

A total of 64 Hox and eight ParaHox genes were identified and their orthology confirmed through Bayesian phylogenetic analysis (Fig. 1). Monophyly of paralog groups *Hox1, Hox2, Lox4, Post1, Post2* and the ParaHox groups *Gsx, Xlox*, and *Cdx* is well-supported (posterior probability > 0.9). Identity of other paralog groups was established by annotating them using information from well-characterised model metazoan and molluscan sequences they cluster with. Supposedly complete (11 genes) or almost complete (nine or more genes) sets of Hox genes were obtained from the polyplacophoran *Acanthochitona crinita*, the neomeniomorphs *Gymnomenia pellucida* and *Wirenia argentea,* as well as the scaphopod *Antalis entalis*. The putatively most incomplete set of Hox genes (three genes) was retrieved from the chaetodermomorph (caudofoveate) *Scutopus ventrolineatus* (Fig. 2).

**Fig. 3:**
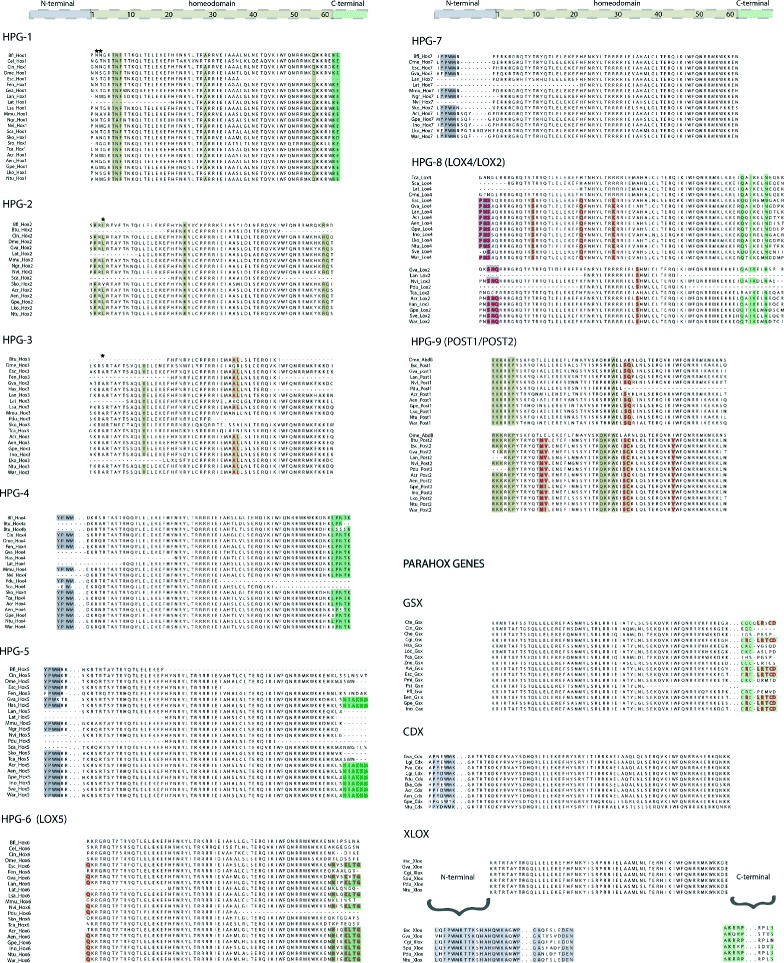
Multiple sequence alignments of Hox and ParaHox sequences highlighting the conserved homeodomain and flanking regions. Bilaterian diagnostic peptides in the homeodomain and in the flanking regions are highlighted by colored boxes. Conserved lophotrochozoan and molluscan residues are highlighted by dark red and green colored letters, respectively. Black stars indicate DNA-contacting residues

The common paralog peptide signatures in the homeobox domain and in its flanking regions greatly differ between the different Hox and ParaHox paralog groups (Fig. 3). The paralog group 1 (HPG-1) contains one conserved motif (positions 6-8) and two unique single amino acid signatures (positions 29 and 56) in the homeobox domain. Additionally, two non-basic amino acids in the N-terminal region inside of the homeobox at positions 2 and 3 (see [54]) and one conserved motif downstream of the homeobox (positions +1 and +2) in the C-terminal region provide unambiguous signatures for the paralog group 1.

The paralog groups 2 (HPG-2) and 3 (HPG-3) have a unique DNA-contacting residue that lies between two conserved basic amino acids at position 4 within the N-terminal region in the homeobox [54]. Furthermore, the paralog group 2 contains three unique single amino acid signatures at position 2, 24, and 58-59, whereas paralog group 3 contains one conserved bilaterian residue at position 14 and one specific lophotrochozoan “AL” motif in the positions 36-37.

The paralog groups 4 (HPG-4) and 5 (HPG-5) do not show any specific motifs or unique residues within the homeodomain. The unique signature of these two paralog groups is the motif “YPWM” located in the upstream N-terminal region outside the homeodomain. Moreover, the paralog group 4 contains a “LPNTK” diagnostic motif in the downstream C-terminal region of the homeodomain (see [55]). A unique specific molluscan motif with 6 residues (“HIAKNM”) was discovered in the paralog group 5, located immediately after the last residue in the C-terminal region of the homeodomain. The paralog group 7 (HPG-7), albeit not possessing a unique signature within the homeodomain, is the only paralog group that contains the region that regulates the Hox-PBC interaction located close to the N-terminal region right before the start of the homeodomain (see [56]).

The Hox sequences belonging to the central class *Lox5* (HPG-6), *Lox2, Lox4* (HPG-8), and the posterior paralog groups *Post-2* and *Post-1* (HPG-9) were characterised by the presence of specific lophotrochozoan signature motifs and amino acid residues in the homeodomain and its surroundings. For example, the presence of a strongly conserved C-terminal parapeptide motif in the paralog genes *Lox5* (Lox5-parapetide)*, Lox2,* and *Lox4* (Ubd-A-parapeptide), and the distinctive homeodomain residues in the paralog genes *Post-1* and *Post-2* (see [57]).

Alignment of the ParaHox genes *Xlox*, *Gbx*, and *Cdx* provides an overview of the conserved homeodomain peptides and the specific signature motifs located in the N- and C-terminal regions of these genes. Among these signature motifs, a specific lophotrochozoan pentapeptide motif (“LRTCD”) in the C-terminal arm of the *Gsx* gene is present. Apart from the gastropod *Lottia* cf. *kogamogai*, the neomeniomorph *Wirenia argentea,* and the chaetodermomorph *Scutopus ventrolineatus*, at least one ParaHox gene was identified in each of the species investigated.

### Gene Ontology and KEGG annotation

**Fig. 4:**
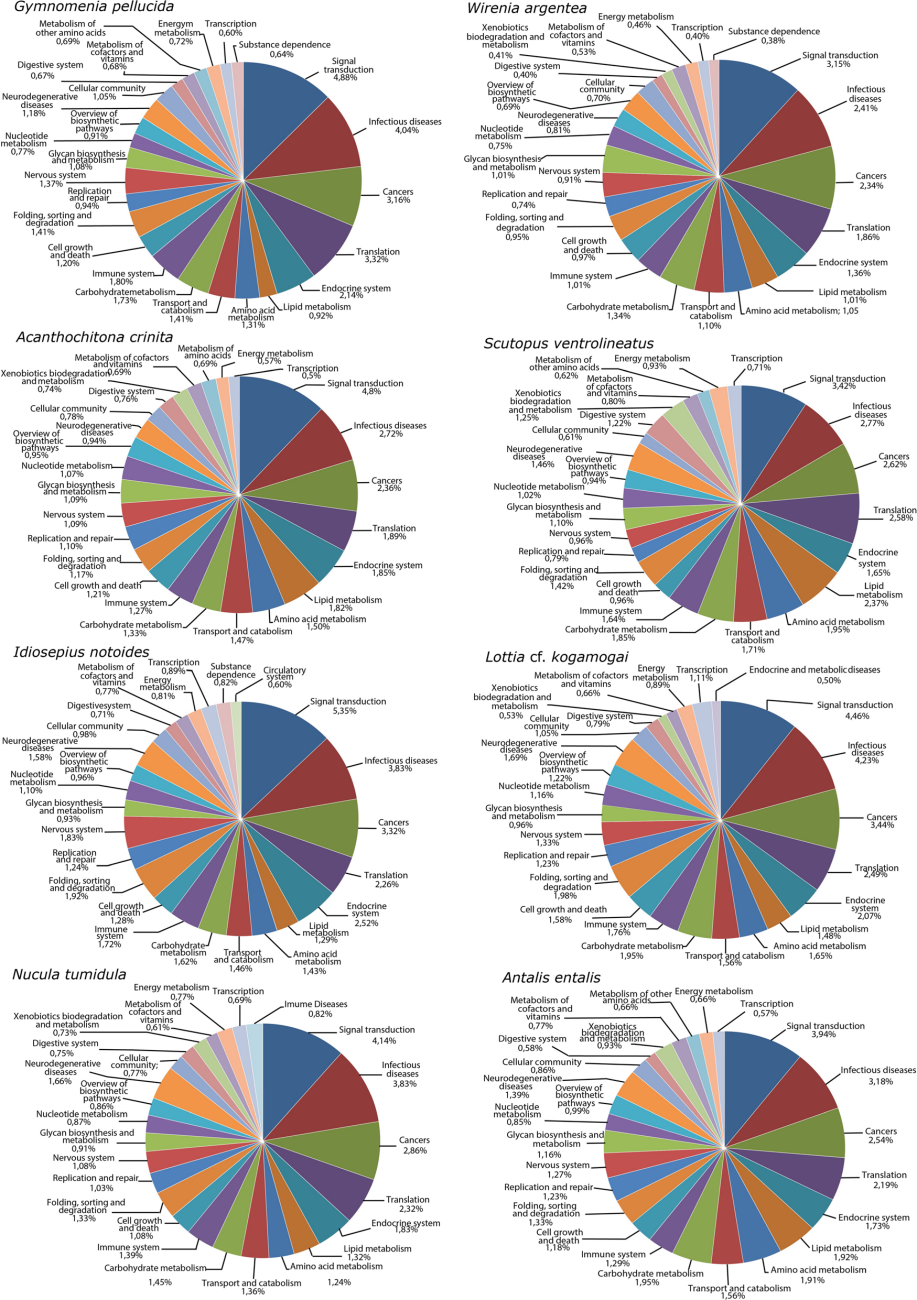
Distribution of the 25 most represented KEGG functional categories in the eight molluscan transcriptomes. The numbers represent the relative percentage of mapped proteins in each category in regard to the total number of transcripts in the respective species

**Fig. 5:**
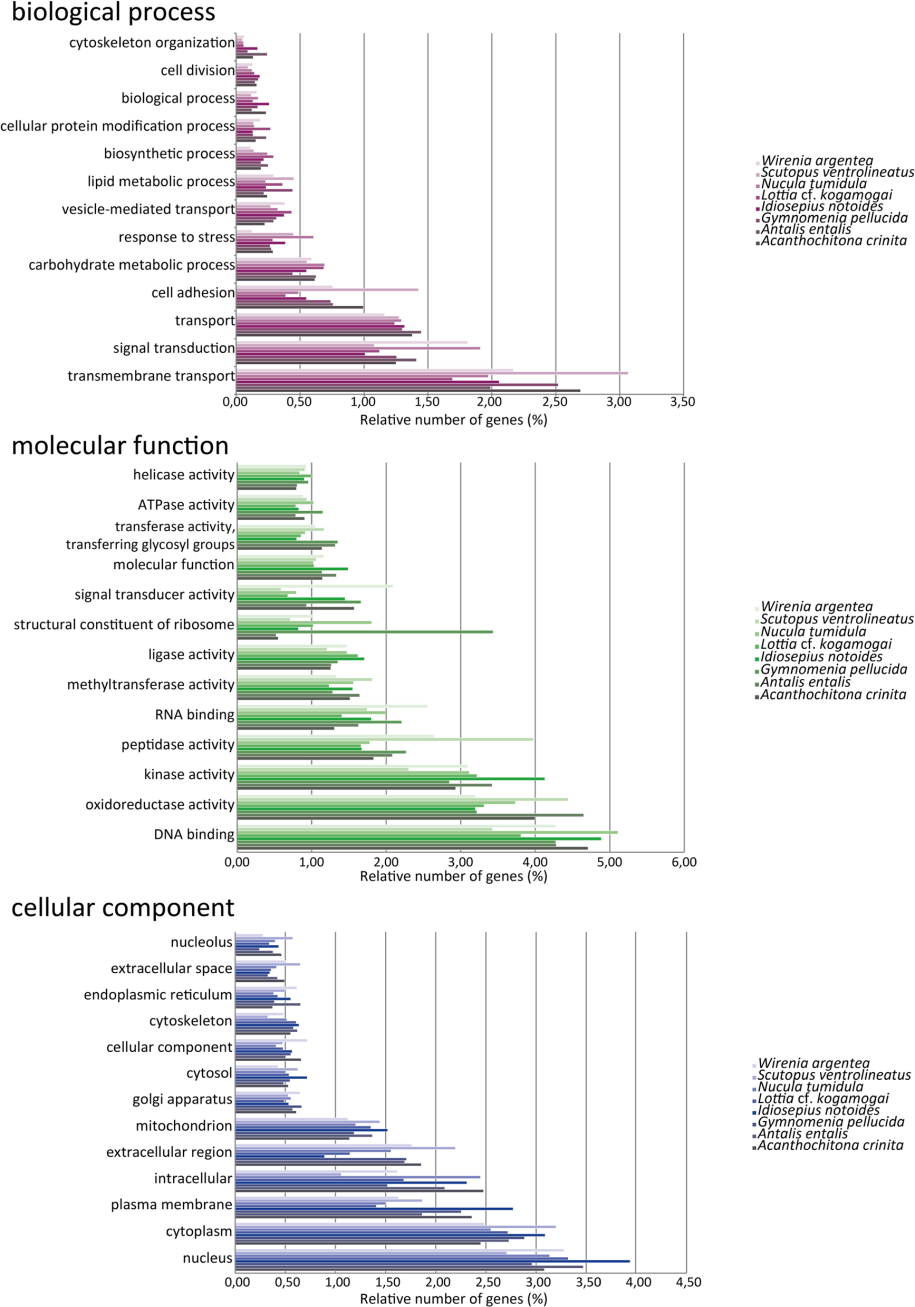
Comparative functional classification using Gene Ontology-Slim terms. Only the 13 most expressed terms in each ontological domain are shown. The relative percentages represent the numbers of mapped GO terms in each category in reads to the total number of transcripts in the respective species

The functional characterisation using the KEGG and Gene Ontology (GO) Slim terms revealed a similar relative percentage of genes distributed in the different functional categories among the molluscan protein gene sets, with a few exceptions (Table 6, Figs. 4 and 5). The percentage of classified proteins belonging to the different molluscan gene sets ranges from 29.59 % to 46.04 % in the KEGG analysis and from 52.0 % to 60.11 % in the GO. Despite the disparity in the number of protein sequences in the molluscan protein gene sets, the number of pathway maps, in which all KEGG Orthology (KO) groups were mapped, is very similar among the species (between 332 and 342).

**Table 6:** Functional annotation of the molluscan transcriptomes using KEGG analysis and Gene Ontology terms

Functional annotation	*Gymnomenia pellucida* (Neomeniomorpha)	*Wirenia argentea* (Neomeniomorpha)	*Scutopus ventrolineatus* (Chaetodermomorpha)	*Acanchothitona crinita* (Polyplacophora)	*Idiosepius notoides* (Cephalopoda)	*Lottia* cf. *kogamogai* (Gastropoda)	*Nucula tumidula* (Bivalvia)	*Antalis entalis* (Scaphopoda)
KEGG (total)	20,861	20,662	16,935	25,460	16,672	9,409	18,524	20,842
Pathways	341	338	340	342	336	332	337	338
Metabolism	3,720	4,324	4,371	5,806	2,951	1,910	3,984	4,958
Genetic Information	2,409	2,180	1,779	2,477	1,834	1,143	2,239	2,146
Enviromental Information	2,087	1,920	1,232	2,443	1,697	809	1,993	1,772
Cellular Processes	1,546	1,636	1,128	1,961	1,128	755	1,433	1,556
Organismal System	2,991	2,655	2,249	3,315	2,609	1,281	2,806	2,557
Human Diseases	3,915	3,619	2,634	3,803	3,127	1,828	4,386	3,367
Not classified	4,193	4,328	3,542	5,655	3,326	1,683	1,683	4,486
GO (total)	23,080	31,149	18,543	30,541	17,469	8,776	23,906	23,779
Biological Process	3,346	4,776	3,196	4,947	2,434	1,352	3,765	3,522
Molecular Function	12,989	16,957	9,683	15,688	9,186	4,662	12,999	12,630
Cellular component	6,745	9,416	5,664	9,906	5,846	2,762	7,142	7,627

**Table 7:** Summary of the sequencing methods, organisms, and mRNA extraction sources

Organism	Class	mRNA source	Sequencing
*Gymnomenia pellucida*	Neomeniomorpha	1/5 total RNA from developmental stages (i.e. freshly hatched larvae until metamorphosis) – 4/5 mRNA from adults.	Illumina
*Wirenia argentea*	Neomeniomorpha	1/7 total RNA from developmental stages (i.e. freshly hatched larvae until metamorphosis) – 6/7 mRNA from adults.	Illumina
*Scutopus ventrolineatus*	Chaetodermomorpha	Total RNA from 2 adult individuals	Illumina
*Acanthochitona crinita*	Polyplacophora	Early cleavage stages – gastrula – early, midstage, and late trochophore larvae – metamorphic competent and settled (post metamorphic) individuals	Illumina
*Idiosepius notoides*	Cephalopoda	Central nervous system of 7 adult individuals	Illumina
*Idiosepius notoides*	Cephalopoda	2/3 total RNA from mixed developmental stages (i.e. stages collected after egg laying until the hatching stage) – 1/3 total RNA from adult central nervous system (brain), arm, and gonads tissue)	454
*Lottia* cf. *kogamogai*	Gastropoda	2/3 total RNA from mixed developmental stages (i.e. trochophore – veliger – pediveliger – metamorphic competent – first juvenile stages) – 1/3 total RNA from adult foot, and central nervous system (CNS)	454
*Nucula tumidula*	Bivalvia	Early cleavage stages – gastrula – early, midstage, and late pericalymma larvae – metamorphic competent and settled (post metamorphic) individuals	Illumina
*Antalis entalis*	Scaphopoda	Early cleavage stages – gastrula – early, midstage, and late trochophore larvae – metamorphic competent and settled (post metamorphic) individuals	Illumina

As expected for transcriptomes sampled from different early developmental stages, the transcriptomes are enriched with proteins that bind and interact with DNA (and thus have, e.g., a putative role in the control of gene expression, chromatin regulation, etc.) and/or RNA (e.g., have a function in RNA processing and modification such as alternative splicing, editing, and polyadenylation). Biological processes involving transmembrane transport as well as carbohydrate and lipid metabolism are overrepresented in relation to other categories in both KEGG and GO analyses. The functional category “signal transduction” is overpopulated with a high relative percentage of proteins in both analyses (between 3-5 % in KEGG and 1–2 % in GO). A deeper look into the fine-grained functional categories inside “signal transduction” in KEGG shows that Notch, Hedgehog, and Wnt are common signaling pathways shared in all gene sets with a high percentage of genes.

**Fig. 6:**
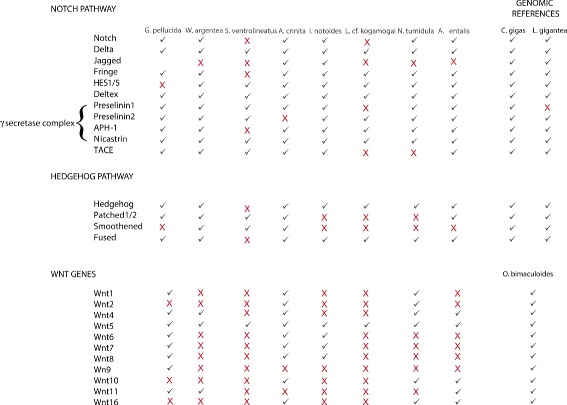
Identification of cardinal gene components in Wnt, Hedgehog, and Notch signaling pathways in the transcriptomes. *Crassostrea gigas*, *Lottia gigantea,* and *Octopus bimaculoides* genes identified from genomic sequences were used for comparison. Not identified sequences are marked by the red “X”

**Fig. 7:**
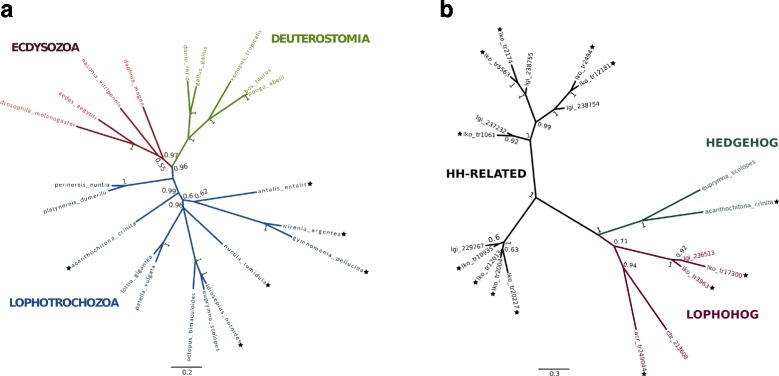
Phylogenetic reconstruction of *Hh* (**a**) and *Hh*-related (**b**) genes from amino acid sequences. The consensus trees were inferred through Bayesian phylogenetic analysis with MrBayes v3.2.2 discarding 25 % of the sampled trees as burn-in. The branch support values are posterior probability values of Bayesian likelihood. Colored branches in A represent the three major superphyla Ecdysozoa, Deuterostomia, and Lophotrochozoa. The Lophohog *Hh*-related family was first described by Bürglin [58] and originally included two sequences retrieved from the lophotrochozoans *Lottia gigantea* (mollusk) and *Capitella teleta* (annelid). Names followed by black stars correspond to newly described sequences obtained in this work

Regarding the Wnt gene family, at least one Wnt gene was found in each of the transcriptomes according to KEGG orthology assignments. The transcriptomes of the aculiferans *Acanthochitona crinita* and *Gymnomenia pellucida* are the most Wnt-rich transcriptomes with nine and eight Wnt representatives, respectively, whereas the gastropod *Lottia* cf. *kogamogai* and the chaetodermomorph *Scutopus ventrolineatus* harbor only the *Wnt5* gene. Additionally, most of the cardinal signaling components of the Notch and Hedgehog pathways were identified and characterised in all transcriptomes, including the *Notch* and *Hh* orthologs (with the exception of *S. ventrolineatus*) (Fig. 6). Phylogenetic analysis with *Hh* genes confirmed the orthology of these molluscan genes and supports the monophyly of the three major clades of bilaterian animals (Deuterostomia, Lophotrochozoa, and Ecdysozoa) (Fig. 7a).

A detailed view at the sequence composition of *Hh* and *Notch* orthologs was performed, highlighting the organisation of the respective protein domains (i.e., N-terminal Hh and C-terminal Hint domain in *Hh* orthologs and EGF-like repeats, LNR, and ANK domains in *Notch* orthologs) and the characteristic conserved residues among these genes. The *Notch* sequences identified herein showed the absence of certain diagnostic motifs in all sequences, with the exception of the protobranch bivalve *Nucula tumidula*, implying that these genes, despite being classified as a *bona fide Notch*, are not represented in their totality (partial coding sequence region). Regarding the *Hh* genes, apart from *Lottia* cf. *kogamogai* in which the Hint domain is missing, all the *Hh* sequences harbor the full length diagnostic Hedgehog domains. Despite the partial *Hh* sequence of the limpet *Lottia* cf. *kogamogai*, the number of *Hh*-related genes retrieved from this transcriptome was the highest among all transcriptomes with 11 representatives. Phylogenetic analysis using the 11 *Hh*-related genes of the limpet, one polyplacophoran *Hh*-related gene obtained from *A. crinita*, and previously published sequences from other lophotrochozoans recovered the “Lophohog” clade, supporting the existence of a lophotrochozoan-specific *Hh*-related gene family ([58]; cf. Fig. 7b).

Overall, the diversity of different GO terms and KEGG functional categories in the protein gene sets show a high resolution picture of the molluscan transcriptomes.

## Discussion

### Feasibility of non-model mollusks for comparative transcriptomic studies

As next-generation sequencing costs have dramatically decreased during the last years, transcriptome shotgun sequencing has emerged as a powerful tool to investigate RNA dynamics of living organisms qualitatively (e.g., which genes are expressed during a given ontogenetic period) and quantitatively (e.g., expression levels of a specific gene) e.g., [59]. Accordingly, it is now feasible to obtain a full catalog of the transcriptome composition and complexity of organisms on a broader and comparative level, enabling to assess several questions in evolutionary biology with the assistance of genomic data [60–62]. Additionally, such a comparative approach is useful to discover shared and unique evolutionary events from different taxa, allowing plausible evolutionary inferences of specific biological questions. The 1KITE (1,000 Insect Transcriptome Evolution) project is a good example as to how next-generation transcriptome sequencing can form the base not only for phylogenetic analyses, but also for insights into genome and transcriptome evolution of species-rich animal clades [63].

In our study, nine new transcriptomes belonging to representatives of seven out of the eight recent class-level taxa of mollusks were deeply sequenced using next-generation sequencing (Illumina and 454). To generate reliable and good quality protein gene sets for downstream analyses (e.g., functional and phylogenetic analyses, sequence identification), various protocols for pre-processing, assembly, clustering, and coding sequence region prediction were established.

Despite many limitations in the *de novo* assembly and the scarce resources of molluscan genomic references (including fully annotated genomes), transcriptome sequencing offers a cost-effective method of characterizing the gene set of non-model species. One challenging aspect in every transcriptomic project is the comparison between assemblies using either common statistics (e.g., N50, number of reconstructed bases, and average length of the transcripts) or annotation-based metrics (e.g., number of single copy orthologs). As pointed out by O´Neil & Emrich [64] and Mundry et al. [65], although many metrics have been used to evaluate and compare these assemblies, it is unclear how precise and accurate these metrics are. Despite these limitations, we assessed and evaluated common statistics in order to compare our assembly results with other recent transcriptome studies on lophotrochozoan organisms (e.g., N50 transcript and number of reconstructed bases). The assembly results obtained herein (excluding the 454 libraries) are at least comparable and in most cases outperform some recent transcriptome studies (cf. [46, 66, 67]). Regarding the completeness and integrity of the transcriptome (i.e. fragmentation of genes), the BUSCO analysis revealed a reasonable completeness in all molluscan libraries, corroborated by the great diversity of gene and gene families identified in the downstream analysis. The high proportion of fragmented genes in the transcriptome of the patellogastropod *Lottia* cf. *kogamogai*, as pointed out by the BUSCO analysis, reflects the high rates of insertions and deletions due to homopolymeric regions during the pyrosequencing process [68], creating frameshifts and disrupting the alignments between these sequences and their respective homologs. Indeed, the first phylogenetic analyses with *Lottia* cf. *kogamogai* Hox genes resulted in atypically long branches showing a great amount of genetic divergence between the patellogastropod sequences and their respective homologs in Mollusca and another bilaterians. Accordingly, even if it remains unclear as to how the aforementioned metrics most accurately reflect the assembly results, comparisons among our data as well as with those of different transcriptome studies clearly demonstrate the high quality of our results.

To date, there are only a few genetic or physical maps publicly available which describe genome organization, extrachromosomal DNA (mitochondrial genomes) [69–71], gene structure, or functional contents for lophotrochozoan animals and especially mollusks. However, three recent studies based on a robust genome annotation in the patellogastropod limpet *Lottia gigantea* [16], the Pacific oyster *Crassostrea gigas* [49], and the octopus *Octopus bimaculoides* [51] have shown that the expected number of protein coding genes in these mollusks ranges from approx. 24,000 to 34,000. In our study, except for *Lottia* cf. *kogamogai* and *Idiosepius notoides*, all protein gene sets have an inflated number of putative proteins when compared to the patellogastropod, oyster, and octopus data. This elevated number of protein-coding genes does not necessarily represent the real complexity of the transcriptomic machinery in our study species; rather, it might be influenced by biases and limitations brought by the next-generation DNA platforms (i.e. fragmentation of genes, sequencing biases) (see [72]) and/or assembly artifacts. Considering the annotation of the coding sequence regions in the different molluscan libraries, a relatively small proportion of proteins (between 21–59 %; see Table 3) have shown sequence homology against well-curated public databases. This high proportion of non-annotated protein sequences is not unusual in transcriptome projects, and this feature is commonly observed in a vast diversity of taxa (cf. [73, 74]), including mollusks [75–77]. This lack of detectable sequence homology in the public databases may be due to several factors, including taxonomically restricted genes (e.g., orphan genes), novel isoform transcripts or protein-coding genes, non-functional coding sequence regions, and poor quality of the sequences themselves or the assembly procedures performed [78, 79]. Specifically in mollusks, various studies have described the emergence of numerous specific suites of genes and gene families, which are either present in different molluscan lineages or are restricted to a single one [51, 80]. The discovery of an independent large-scale expansion and evolution of the tyrosinase gene family in bivalves [81] is a good example of how comparative genomics and transcriptomics are useful to characterise novel lineage-specific genes and gene families.

### Diversity of Hox and ParaHox genes in mollusks

To elucidate the utility of the molluscan transcriptomes for evo-devo studies, an extensive comparative survey was conducted focussing on Hox and ParaHox gene sequences. A total of 64 Hox and eight ParaHox genes were found and fully characterised. Prior to our study, complete (or near-complete) sets of Hox genes had only been identified in three bivalve species (*Pecten maximus, Crassostrea gigas*, and *Pinctada fucata*) [49, 82, 83], two marine gastropods (*Gibbula varia* and *Lottia gigantea*) [16, 38], and in two cephalopods (the squid *Euprymna scolopes* and the octopod *Octopus bimaculoides*) [51, 84]. We here report a complete Hox gene complement for the neomeniomorph *Gymnomenia pellucida*. Additionally, at least near-complete Hox gene complements were identified from the polyplacophoran *Acanthochitona crinita*, (10 genes), the scaphopod *Antalis entalis* (10 genes), and another neomeniomorph, *Wirenia argentea* (nine genes). Notably, only few ParaHox sequences were retrieved from our molluscan transcriptomes, considering that all three ParaHox genes had been found in various molluscan lineages prior to our analysis [16, 42, 49, 83, 85].

The publicly available genomic resources and the data presented here show that the molluscan Hox and ParaHox clusters share a similar composition in terms of gene content despite the great disparity of morphological features within the phylum [26]. This implies that the rich morphological diversity among different class-level taxa of mollusks lies in the regulation and subtle changes of the regulatory networks in the developmental program rather than in the physical organisation and composition of the Hox and ParaHox clusters. By comparing Hox sequences from a vertebrate, fly, and amphioxus, it was proposed earlier that many of the amino acid replacements used as diagnostic criteria for the different paralog groups are likely to be localised on the surface of the respective proteins and have a major functional impact on protein-protein interactions [86]. This fact, associated with the relaxed DNA-binding specificity of the homeodomain, provides the necessary toolbox to promptly originate new regulatory interactions between the Hox genes and their target genes [87], thereby forming an important prerequisite for the evolution of novel morphological features. Within Mollusca, a striking example as to how the possible relaxation of the regulatory constraints and the recruitment of novel regulatory genes are responsible for morphological changes has been reported for the cephalopod *Euprymna scolopes* [36]. Hox gene expression in this bobtail squid deviates from the proposed ancestral role of patterning the antero-posterior body axis; instead, the reported Hox genes are expressed during ontogeny of various taxon-specific morphological innovations such as the brachial crown, funnel, light organ, or the stellate ganglia. In addition to the striking plasticity of the Hox genes and their functional co-option during evolution, the study also proposed the possibility that the non-collinear mode of expression of these genes in cephalopods correlates with the disruption of the Hox cluster in the genome. This notion has recently been confirmed by detailed analyses of the genome of an octopod [51]. Concerning the ParaHox genes, it was shown that the expression of *Gsx* in the gastropod *Gibbula varia* coincides with the area that surrounds the radula anlage, indicating that the function of this homeobox gene was co-opted into the formation of this molluscan autapomorphy [42]. Studies on a scaphopod and the pygmy squid *Idiosepius*, however, revealed a different scenario, whereby *Gsx* is expressed in components of the developing larval and adult nervous system, respectively, but not in the digestive tract or the developing radula, thus again demonstrating the plasticity of Hox and ParaHox expression domains across Mollusca [43].

It is difficult to determine whether the lack of specific Hox and ParaHox genes in the species of our study is due to gene loss, methodological biases, or low gene expression levels. However, loss of certain genes in both Hox and ParaHox clusters has been described as a recurrent event in the evolutionary history of metazoans [16, 88, 89] including mollusks [45, 49, 90]. Tunicates are a prime example as to how massive gene losses and disrupture of the cluster-like chromosomal organisation can occur in the Hox gene complement [91]. As such, disintegration of the Hox cluster and the loss of central class Hox genes have been reported for the tunicates *Oikopleura dioica* [92] and *Ciona intestinalis* [93]. Losses involving the anterior, central, and posterior Hox as well as the ParaHox genes have been shown by whole genome sequencing studies in cephalopods and bivalves [49, 51]. In addition, various molecular studies have failed to amplify and retrieve particular Hox and ParaHox gene fragments from a wide range of molluscan lineages [45, 84, 90, 94]. Taking into consideration these gene losses, the high degree of completeness of the scaphopod and polyplacophoran transcriptomes obtained from our BUSCO searches (94.54 % and 94.42 %, respectively) and the deep transcriptome sequencing, it seems reasonable to assume that both the polyplacophoran (*Acanthochitona crinita*) and the scaphopod (*Antalis entalis*) Hox set are made up of 10 genes and are represented in their totality in our analysis. Regardless of the Hox and ParaHox set completeness, it is important to notice that the Hox and ParaHox sequences identified in this study contain the full length protein-coding sequence and are long and informative enough for a great deal of molecular (e.g., *in situ* hybridisation) and bioinformatics applications (e.g., phylogenetic analysis).

The identification and characterisation of signature residues (i.e., residues that are shared at certain positions by orthologous proteins but not likely to be present in paralogous proteins) inside the homeodomain and in the surroundings of N-terminal and C-terminal regions provides a better understanding of the evolutionary history of Hox genes [7] and metazoan phylogeny. Herein, a hexapeptide molluscan motif in the paralog group 5 is described for the first time, together with a lophotrochozoan five residue motif in the C-terminal arm of the ParaHox gene *Gsx*. The molluscan-specific motif represents an important marker in distinguishing, from the same paralog, closely related species. To date, this is the first molluscan-specific motif related to a Hox paralog group. These findings show the suitability of the molluscan transcriptomes for the identification of target developmental genes and the specific fine-grained characterisation of these sequences in a phylogenetic context.

Recently, two phylogenomic studies have shed light on the evolutionary interrelationships between seven [60] or the entire eight recent class-level taxa of Mollusca ([61]; see also [95] for some corrections of their 2011 analysis). Remarkably, both analyses strongly support the Aculifera-Conchifera hypothesis, i.e., a basal split of Mollusca into a clade comprising all mollusks that derive from an ancestor with a single shell (Conchifera) and a taxon uniting both aplacophoran clades (Neomeniomorpha and Chaetodermomorpha) with the Polyplacophora as Aculifera. In the light of these results, the characterisation of the Hox and ParaHox gene sets described herein, which includes four aculiferan species, provides an important prerequisite for gene expression studies, and thus research into assessing the putative functional plasticity of these genes across Mollusca. As a matter of fact, expression patterns of ten Hox (all representatives except *Post-1*) and one ParaHox gene (*Cdx*), based on the transcriptome of the polyplacophoran *Acanthochitona crinita* analysed herein, have recently become available from our group [41, 44]. These studies show that the Hox genes in polyplacophorans are expressed in a conserved anterior-posterior pattern along the primary (i.e., longitudinal) body axis. Thereby, their expression was found to be staggered and not restricted to trochozoan- or molluscan-specific features such as the prototroch, the apical organ, or the anlagen of the shell (plates). Instead, the Hox genes are expressed in contiguous domains originating from different germ layers. This is in stark contrast to cephalopod and gastropod mollusks, where they are expressed in a non-staggered fashion in the foot, apical organ [35, 37, 38, 96] or in taxon-specific features of the squid *Euprymna* [36]. Thus, the polyplacophoran Hox gene expression pattern is more similar to annelids than to their molluscan allies. This has led to the conclusion that the Hox genes were co-opted into the patterning of morphological novelties in at least some conchiferans, a situation that most likely contributed to the evolutionary successes of its representatives (see [26]).

### Functional characterisation and diversity of the gene repertoire in mollusks

The ability to correlate individual sequences and their respective molecular function is an important step to elucidate the biological background of large numbers of genes (e.g., a putative role in axis specification, neurogenesis, digestive tract formation, and the like). The categorisation of genes and gene products into well-constructed hierarchical classes and pathways aids in the understanding of both cell and organismal biology [97, 98]. This use of molecular information also aids in understanding genetic regulatory networks that control expression levels of mRNA and proteins. The GO as well as KEGG enrichment analyses showed a common overlap of functional categories, which are compatible with the biological background where the transcriptomes were sampled. The functional GO terms “DNA binding”, “nucleus”, and “methyltransferase activity” are terms with a high relative percentage of proteins in all gene sets. This reflects the transcriptome background during the development of the species, composed by the presence of many proteins involved in the basal regulation of the transcription (e.g., general transcription factors), development (e.g., homeobox genes such as Hox and ParaHox genes), and protein methylation (e.g., regulation of the epigenetic levels that affect transcription).

Considerable differences were found between the KEGG categories and GO terms retrieved from the predatory sea snail *Rapana venosa* (larval and post-larval stages) [99] and the transcriptomes presented herein. The number of different metabolic pathways into which the proteins were mapped was also found to be higher in our study (between 332 and 342 pathways) than in that of Song et al. [99] (270 pathways). However, this discrepancy can be explained by the nature of the biological samples used to construct the RNA libraries. While the six *R. venosa* samples consisted of only larval and post-larval stages, our samples covered larval, post-larval, juvenile, and adult stages. Due to this broader sampling, one would expect a higher number of metabolic pathways in our analysis than in that of Song et al. [99]. The low percentage and absence of some developmental genes in the *Scutopus ventrolineatus* and *Lottia* cf. *kogamogai* transcriptomes, as revealed by the functional analysis with KEGG and GO, as well as the Hox and ParaHox survey, is a direct reflection of the use of adult specimens during the construction of the transcriptome library and the shallow depth of the 454 sequencing methodology, respectively.

The Wnt, Hedgehog, and Notch signaling pathways are related to the regulation of cell proliferation, transcription, translation, and the proper embryonic development of bilaterian animals, in which any interruption of their signaling activity has severe consequences on developmental outcomes [100]. Thirteen Wnt subfamilies have been characterised in metazoans, while lophotrochozoan representatives, such as the polychaete annelids *Capitella teleta* and *Platynereis dumerilli,* commonly possess only 12 subfamilies and the basal-branching gastropods *Patella vulgata* and *Lottia gigantea* only nine (*WntA*, *Wnt1*, *Wnt2*, *Wnt4*, *Wnt5*, *Wnt6*, *Wnt7*, *Wnt9,* and *Wnt10*) [101, 102]. We found three additional subfamilies in mollusks using KEGG orthology assignment, namely *Wnt8*, *Wnt11,* and *Wnt16*, suggesting that molluscan gene content in the Wnt subfamilies matches that of their lophotrochozoan relatives. Indeed, in a recent publication of the genome of the cephalopod *Octopus bimaculoides* [51], the presence of 12 Wnt genes was reported, corroborating our results and expanding the Wnt complement to at least 12 genes in Mollusca. The *Wnt3* gene is not present in any molluscan transcriptome analysed so far and is likewise absent in all other lophotrochozoans and ecdysozoans hitherto examined (but not in cnidarians) (see [103, 104]), reinforcing the idea that this gene was lost at the base of Protostomia.

Regarding the Hedgehog and Notch signaling pathways, no study focusing on the characterisation and phylogenetic relationships of these genes in mollusks is currently available. The limited knowledge about these important pathways is restricted to some gene expression studies in a few gastropod and cephalopod representatives [105, 106]. Comparisons with respect to the core components present in these two pathways between the transcriptomes described here and two molluscan reference genomes (the limpet *Lottia gigantea* and the oyster *Crassostrea gigas*) revealed a highly shared molecular framework. These results are not surprising, given that both signal transduction pathways play a fundamental role in animal development (e.g., patterning of body axes) and have been characterised in several metazoan animals, from sponges [107] to chordates including humans [108]. Our analysis of the domain organisation in *Notch* and *Hh* orthologs revealed different architectures and patterns of conservation within mollusks and other major groups of bilaterian animals (ecdysozoans and deuterostomes). The receptor Notch is a multidomain protein made by six different components: 30 to 40 amino acids EGF (epidermal growth factor) repeats containing six conserved cysteines; three LNR (lin-notch-repeat) or Notch domains; one NOD and NODP domain; a RAM 23 domain; a PEST domain; and, finally, several Ankyrin repeats [109]. Comparisons of the EGF domain content between the basally-branching bivalve *Nucula tumidula*, the polyplacophoran *Acanthochitona crinita*, and the gastropod *Lottia gigantea Notch* sequences revealed the presence of 34 to up to 36 repeats in these lophotrochozoan proteins. The presence of the NOD and NOPD domains has also been reported for the bivalve *N. tumidula* and is shared by the gastropod *L. gigantea*. The function of these domains is still obscure and remains to be elucidated, albeit they are present in almost all major metazoan lineages (with the exception of the Porifera) [110].

The *Hh* gene family is present throughout the Metazoa, being secondarily lost in some lineages. For example, the nematode *Caenorhabditis elegans* lacks an *Hh* ortholog, whereas *Drosophila melanogaster*, the sea anemone *Nematostella vectensis*, and mammals have one, two, and three *Hh* genes, respectively [111–114]. Herein, one single *Hh* gene was identified in each of the molluscan transcriptomes (apart from *Scutopus ventrolineatus*) through KEGG orthology assignments. Notably, a distinct *Hh*-related family named “Lophohog” was previously retrieved from the genomes of the annelid *Capitella sp. I* and the gastropod *Lottia gigantea* [58]. In this study, 12 *Hh-*related genes were first identified and described for the basally branching gastropod *Lottia* cf. *kogamogai* (11 genes) and the polyplacophoran *Acanthochitona crinita* (one gene)*.* No *Hh*-related genes were found in the remaining transcriptomes analysed in this study. Interestingly, three new *Hh*-related sequences (two from the limpet *L.* cf. *kogamogai* and one from the polyplacophoran *A. crinita*) showed a close relationship with Lophohog members, expanding the previously described Lophohog clade to five sequences. Accordingly, it seems that the genomes of the basally branching gastropods *L.* cf. *kogamogai* and *L. gigantea* are enriched with *Hh-*related genes, more than in any other molluscan representative investigated to date. The apparent lack of Lophohog representatives in the other mollusks investigated herein must be treated with care as it may not represent the real genetic background as fixed in the genome of these species due to the nature of the transcriptome sequencing; however, the available genomic and transcriptomic data so far support such a scenario. It is expected that the evolution of *Hh* and *Hh*-related sequences will become clearer as soon as additional molluscan genomes become available.

## Conclusions

Mollusks show a striking diversity of body plans and are a key taxon for a better understanding of the underlying mechanisms that guide the evolution of developmental processes in multicellular animals. In this study, high-quality transcriptomes were generated from eight molluscan species, representing seven of the eight recent class-level taxa. Different pipelines were carefully designed and implemented, yielding results that are comparable with those generated from model organisms. Furthermore, an extensive catalog of annotated gene products was generated for application in a broad range of downstream analyses. The study focused on the identification and evolution of important developmental genes (Hox and ParaHox) and molecular pathways, nevertheless the results can be used in a broad range of *in silico* (e.g., phylogenomics and gene profiling) and molecular developmental and functional analyses (e.g., *in situ* localisation of mRNAs, expression and characterisation of cloned genes, gene silencing). The data presented herein increase the knowledge on the molecular toolkit of mollusks, especially of the understudied aplacophoran clades, and provides a valuable molecular resource, in particular for further research with a focus on comparative evolutionary developmental (i.e., evo-devo) studies.

## Methods

### Collection sites, animal cultures, RNA extraction, and fixation

Adults of the polyplacophoran *Acanthochitona crinita* were collected at the Station Biologique de Roscoff, (Roscoff, France) during the summers of 2013 and 2014. Embryos were cultured and staged as previously described [41, 115]. Several hundred individuals of early cleavage stages, blastulae, gastrulae, trochophore larvae, and metamorphic competent individuals as well as early juveniles were collected. Adults of the solenogasters (= neomeniomorphs) *Wirenia argentea* and *Gymnomenia pellucida*, the basally branching protobranch bivalve *Nucula tumidula*, and the caudofoveate (= chaetodermomorph) *Scutopus ventrolineatus* were collected from sediment that was sampled with a hyperbenthic sled at 180–220 meter depth on muddy seafloor in Hauglandsosen (Bergen, Norway) during the winters of 2012 and 2013. The solenogaster and bivalve embryos were cultured and staged as previously described [28, 115, 116]. Adults of *S. ventrolineatus* were kept at 6.5 °C in UV-treated millipore-filtered seawater (MFSW) at the marine living animal facilities at the Department of Biology, University of Bergen, and total RNA of two adult individuals was extracted. Several hundred individuals of early cleavage stages, blastulae, gastrulae, pericalymma (i.e., test cell) larvae, and metamorphic competent as well as juvenile individuals were collected from the solenogaster and bivalve species. Adults of the scaphopod *Antalis entalis* were collected from approx. 30 m depth by the staff of the research vessel *Neomys* off the coast of Roscoff (France). Embryos were cultured and staged as previously described [43]. A total of several hundred individuals of mixed developmental stages up to the early juveniles were collected. Adults of the pygmy squid *Idiosepius notoides* were dip-netted in the sea grass beds of Moreton Bay, Queensland, Australia. Adult squids were kept in closed aquaria facilities at the School of Biological Sciences of the University of Queensland and the RNA of seven nervous systems of adults was collected. Embryos of *I. notoides* were cultured and staged as previously described [117]. Several individuals (approx. 300) representing all stages from freshly laid zygotes to hatchlings were collected. Adults of the basally branching patellogastropod *Lottia* cf. *kogamogai* were collected from intertidal rocks and stones in the vicinity of the marine biological station Vostok (approx. 150 km north of Vladivostok, Russian Federation). Embryos and adults of *L.* cf *kogamogai* were cultured and staged as previously described [118, 119]. Several hundred *L.* cf. *kogamogai* embryos, larvae, and juveniles of key developmental stages (i.e. trochophore, veliger, metamorphic competent, early juvenile stages) were collected.

For RNA extraction, some individuals were stored in RNAlater (Lifetechnologies, Vienna, Austria) at −20 to −80 °C. The RNA of these specimens as well as freshly collected specimens was extracted with a Qiagen extraction kit (Roermond, Netherlands) and subsequently stored at −80 °C. Representatives of the cryptic monoplacophorans were not accessible to us for this study.

### Next-generation sequencing, sequence pre-processing, and filtering

High-quality molluscan transcriptome libraries using next-generation sequencing were generated for each one of the aforementioned class-level taxa (Table 7). The short-read libraries were generated with Illumina HiSeq 2000, chemistry v3.0, 2 x 100 pb paired-end modules and the normalised random-primed cDNA. 454 libraries were generated with GS FLX+ with read length of up to 750 bp. The number of reads (or read pairs in Illumina libraries) generated per pooled transcriptomic library varied between 402,814 (*Lottia* cf. *kogamogai*) and 53,751,440 (*Gymnomenia pellucida*), depending on the sequencing technology used.

To remove low quality reads and avoid substandard results in the downstream analyses, different pre-processing bioinformatics pipelines were developed and empirically tested regarding the sequencing method used to obtain the transcriptomic libraries. The short-read libraries preprocessing (Illumina) was carried out using the multithreaded command line tool trimmomatic v0.3.2 [120]. Known specific Illumina adapters were removed from the paired-end libraries with the parameter ILLUMINACLIP:adapters/TruSeq3-PE-2.fa:2:30. The filtering by quality and length was executed with the command line SLIDINGWINDOW:4:15 MINLEN:40 for all the transcriptomes except for the *Wirenia argentea* library, in which the parameters SLIDINGWINDOW:4:20 MINLEN:40 were defined. The long read libraries (454) were trimmed and converted from SFF (Standard Flowgram Format) to fasta and fasta.qual with the program sff_extract.py v0.3.0 included in the seq_crumbs package (http://bioinf.comav.upv.es/seq_crumbs/) with the default parameters as well as -min_left_clip = 30 parameter for *Lottia* cf. *kogamogai* and –min_left_clip = 32 for the *Idiosepius notoides* library. The quality of the filtered libraries was assessed with the software fastx_toolkit (http://hannonlab.cshl.edu/fastx_toolkit/) taking into consideration the quality score of the bases, the GC-content, and the read length. The assemblies and all downstream analyses were conducted with high-quality and clean libraries.

### Transcriptome assembly and quality assessment

The filtered short-read and long-read transcriptome libraries were reconstructed into contiguous cDNA sequences with IDBA_tran v1.1.1 [121] and MIRA4 [122] software, respectively. Information regarding the mRNA sources is summarised in Table 7. The short-read transcriptome assemblies with IDBA_tran were executed with the parameters –mink 20 –maxk 60 –step 5, except for the *Wirenia argentea* library for which the additional parameter –max_count 3 was used. All long-read transcriptome assembling was executed with the parameter mmhr = 2 and the default settings. The quantitative quality assessment of the reconstructed libraries were carried out using QUAST program v2.3 [123] regarding the number of transcripts, number of total bases reconstructed, N50 value, and GC content. The assembling results of the different *Idiosepius notoides* libraries (454 and Illumina) were combined and used in all posterior downstream analyses.

### Identification of the coding sequence regions (CDS)

To predict the most probable coding sequence regions within the transcripts, an empirical homology-based methodology was designed using Novaes et al. [124] as a guide, rather than the use of gene prediction tools. The use of gene prediction tools requires the construction of a high-quality training dataset, an arduous task for understudied animals as those used herein. All the reconstructed sequences were translated into protein sequences (located between a start and a stop codon) greater than 50 amino acids in length with the program getorf from the EMBOSS package (http://emboss.sourceforge.net/). The libraries were then submitted to similarity searches with a defined e-value of 1e-06 against three well-curated reference libraries (Uniref90, Pfam and CDD) using the blastp [125], hmmsearch [126], and rps-blast [125] tools, respectively. An in-house Perl script was written in order to select the unique CDS in each transcript with the highest number of evidences (positive hits against the reference library). All the posterior downstream analyses were conducted with the protein gene set libraries created with the aforementioned procedure.

### Generation of molluscan non-redundant gene sets

To decrease the computation resources required for the downstream analyses and prevent inflation of the results, the redundancy of the molluscan protein gene sets was reduced using the program UCLUST [127]. The protein sequences with 100 % identity were clustered together, in which the identity is a measure of the number of matches (identities) between two sequences divided by the number of alignment columns.

### Assessment of completeness of protein gene sets

In addition to the statistical assessment of the assembled transcriptomes (e.g., N50 values, number of reconstructed base pairs), an analysis to assess the protein gene set completeness in terms of gene content was performed, in order to provide a better understanding and interpretation of the results obtained in the downstream analyses. The assessment of gene content and completeness of the protein gene sets was performed with the program BUSCO using the pre-defined metazoan Benchmarking set of Universal Single-Copy Orthologs with 843 evolutionary conserved orthologous groups [53]. The gene sets were classified into BUSCO metrics as follows: C: complete, D: duplicated, F: fragmented, M: missing.

### Hox and Parahox sequence identification and phylogenetic analysis

The protein libraries from all transcriptomes were used in local similarity searches using the program blastp [125] against known and well-curated molluscan Hox and ParaHox sequences retrieved from GenBank non-redundant protein database. The top 3 blast hits of each similarity search were analysed and re-blasted against the entire GenBank non-redundant protein database to reconfirm the homology. Additionally, each putative Hox and ParaHox gene was independently aligned together with their representative homologs from several different metazoan phyla also retrieved from GenBank non-redundant database using the program mafft [128] with the parameters –max_iterate 1000 –localpair. The multiple sequence alignment containing the Hox and ParaHox sequences were searched for the presence of the diagnostic residues/motifs in the homeodomain as well as in the flanking regions. Frameshift errors in *Lottia* cf. *kogamogai Hox1*/*Hox2*/*Post-1*/*Post-2* sequences were corrected using the HMM-FRAME program [129]. All the sequences were then manually edited with the program aliview [130]. The phylogenetic analysis was carried out using MrBayes v3.2.6 [131] with Jones-Taylor-Thornton model of amino-acid substitution [132] as determined using Akaike information criterion (AIC) as implemented in prottest3 [133], 6 rates categories for the gamma distribution, and 30,000,000 generations. After the removal of the initial 25 % of the sampled trees as burn-in, the quality of the run was assessed using Tracer (http://beast.bio.ed.ac.uk/Tracer), regarding the convergence of the likelihood values. The final phylogenetic tree was created and edited with Figtree (http://tree.bio.ed.ac.uk/software/figtree/). The list of species and gene names, phyla, and GenBank accession numbers used in the phylogeny are available in Additional file 1: Table S1.

### Identification of *Hh* and *Hh*-related genes and phylogenetic analysis

The *Hh* and *Hh*-related genes were retrieved from the molluscan transcriptomes based on the KEGG orthology assignments. All putative sequences were blasted against known protein databases (PFAM, CDD, and the non-redundant protein database from NCBI), in order to reconfirm the initial orthology assignments. The *Hh* and *Hh*-related sequences were aligned, edited, the phylogeny inferred, and the final tree generated as described above. Frameshift errors in *Lottia* cf. *kogamogai* lko_tr2004/lko_tr12013/lko_20227 were corrected using HMM-Frame program [129]. The substitution model, the number of generations, and sample frequency defined in MrBayes were WAG + G model of amino acid substitution [134], 30,000,000, and 1,000 respectively. The list of species and gene names, phyla, and GenBank accession numbers of the sequences used in the phylogeny are available in Additional file 2: Table S2 and Additional file 3: Table S3.

### GO-Slim annotation and pathway mapping with KEGG

The Gene Ontology analyses (GO) were performed in two steps. First, all protein databases that originated from the high-quality assembled transcriptomes were locally blasted against the UniProtKG database. In the second step all transcripts with positive GO-ids were categorised and quantified (with an in-house Perl script) into the generic 149 categories of the GO-Slim database (http://www.ebi.ac.uk), including the three main ontologies: biological process, cellular component, and molecular function.

The KEGG analysis was performed online through KAAS (KEGG Automatic Annotation Server) using the bi-directional best hit (BBH) methodology and the Gene database. First, all proteins were annotated using the KEGG GENES ortholog group database. This procedure assigned KO (Kegg Orthology) identifiers to the proteins, which were then mapped to BRITE hierarchies of functional classifications. The KEGG results were then categorised and quantified with the help of an in-house Perl script.

## Additional files

Additional file 1: Table S1. Data used for the phylogenetic analysis of Hox and ParaHox genes, including the respective GenBank accession numbers. (DOC 31 kb)
Additional file 2: Table S2. Data used for the phylogenetic analysis of *Hedgehog* genes including the respective GenBank accession numbers. (DOC 31 kb)
Additional file 3: Table S3. Data used for the phylogenetic analysis of *Hh*-related genes including the respective GenBank accession numbers. (DOC 31 kb)

## Abbreviations

AIC: Akaike information criterion
bp: Base pairs
BBH: Bi-directional best hit
BLAST: Basic local alignment search tool
BUSCO: Benchmarking set of Universal Single-Copy Orthologs
CDD: Conserved domain database
CDS: Coding sequence region
CNS: Central nervous system
DNA: Deoxyribonucleic acid
EGF: Epidermal growth factor
E-value: Expect value
GO: Gene ontology
HH: Hedgehog
HMM: Hidden Markov model
HPG: Hox paralog group
KAAS: KEGG automatic annotation server
KEGG: Kyoto encyclopedia of genes and genomes
KO: Kegg orthology
LNR: Lin-notch-repeat
NCBI: National Center of Biotechnology Information
PEST domain: Proline-glutamic-acid-serine-threonine domain
RNA: Ribonucleic acid
SFF: Standard flowgram format.
