# Supplementary material for: Comparative transcriptomics enlarges the toolkit of known developmental genes in mollusks
Source: BMC Genomics. 2016 Nov 10;17:905. doi: 10.1186/s12864-016-3080-9 (PMC5103448; doi:10.1186/s12864-016-3080-9)
Supplement: Additional file 1: Table S1. — Data used for the phylogenetic analysis of Hox and ParaHox genes, including the respective GenBank accession numbers. (DOC 31 kb) [file 12864_2016_3080_MOESM1_ESM.doc]

**Additional file 1: Table S1 Data used for the phylogenetic analysis of Hox and ParaHox genes, including the respective GenBank accession numbers.**

| ***Species name*/Abbreviation** | **Phylum** | **Gene Name** | **Accession Number** |
| --- | --- | --- | --- |
| *Acanthochitona crinita* / Acr | Mollusca | *AcrHox1*  *AcrHox2*  *AcrHox3*  *AcrHox4*  *AcrHox5*  *AcrLox5*  *AcrHox7*  *AcrLox4*  *AcrLox2*  *AcrPost2*  *AcrCdx* | KX365078  KX365079  KX365080  KX365081  KX365082  KX365083  KX395148  KX365085  KX365084  KX365086  KX365087 |
| *Antalis entalis* / Aen | Mollusca | *AenHox1*  *AenHox2*  *AenHox3*  *AenHox4*  *AenHox5*  *AenLox5*  *AenLox4*  *AenLox2*  *AenPost1*  *AenPost2*  *AenCdx*  *AenGsx* | KX365088  KX365089  KX365090  KX365091  KX365092  KX365093  KX365095  KX365094  KX365096  KX365097  KX365098  KX365099 |
| *Branchiostoma floridae* / Bfl | Chordata | *BflHox1*  *BflHox2*  *BflHox4*  *BflHox5*  *BflHox6*  *BflHox7* | BAA78620  BAA78621  BAA78622  ABX39489  ABX39490  ABX39491 |
| *Bugula turrita* / Btu | Bryozoa | *BtuHox2*  *BtuHox3*  *BtuHox4a*  *BtuHox4b*  *BtuPost2* | AAS77225  AAS77226  AAS77227  AAS77228  AAS77230 |
| *Caenorhabditis elegans* / Cel | Nematoda | *CelHox1*  *CelHox6* | CAA34929  AAA28106 |
| *Capitella teleta* / Cte | Annelida | *CteGsx* | AAZ23124 |
| *Ciona intestinalis* / Cin | Chordata | *CinHox1*  *CinHox2*  *CinHox4*  *CinHox5*  *CinHox6*  *CinGsx* | NP_001122333  CAD59668  NP_001027781  NP_001027665  CAD59670  NP_001027663 |
| *Clytia hemisphaerica* / Che | Cnidaria | *CheGsx* | ACM62729 |
| *Crassostrea gigas* / Cgi | Mollusca | *CgiXlox*  *CgiCdx*  *CgiGsx* | XP_011426134  XP_011420260  XP_011426138 |
| *Drosophila melanogaster* / Dme | Arthropoda | *DmeHox1*  *DmeHox2*  *DmeHox3*  *DmeHox4*  *DmeHox5*  *DmeHox6*  *DmeHox7*  *DmeHox8*  *DmeDll*  *DmeEng* | CAB57787  CAA45271  P09089  P07548  NP_524248  NP_477498  CAA27417  CAA29194  NP_726486  P02836 |
| *Euperipatoides kanangrensis* / Eka | Onychophora | *EkaCdx* | CEP25538 |
| *Euprymna scolopes* / Esc | Mollusca | *EscHox1*  *EscHox3*  *EscHox5*  *EscHox7*  *EscLox4*  *EscPost1*  *EscPost2*  *EscXlox*  *EscGsx* | AAL25804  AAR16188  AAR16189  AAL25809  AAL25810  AAL25811  AAL25812  ABD16192  AAV85466 |
| *Flaccisagitta enflata* / Fen | Chaetognatha | *FenHox1*  *FenHox3*  *FenHox4*  *FenHox5*  *FenHox6*  *FenHox8*  *FenPost1*  *FenPost2* | ABS18809  ABS18810  ABS18811  ABS18812  ABS18813  ABS18814  ABS18815  ABS18816 |
| *Gibbula varia* / Gva | Mollusca | *GvaHox1*  *GvaHox2*  *GvaHox3*  *GvaHox4*  *GvaHox5*  *GvaLox5*  *GvaHox7*  *GvaLox4*  *GvaLox2*  *GvaPost1*  *GvaPost2*  *GvaCdx*  *GvaXlox* | ACX84671  ADJ18233  ADJ18232  ACX84672  ADJ18234  ADJ18235  ADJ18235  ADJ18237  ADJ18238  ACX84673  ACX84674  ALM30866  ADJ18240 |
| *Gymnomenia pellucida* / Gpe | Mollusca | *GpeHox1*  *GpeHox2*  *GpeHox3*  *GpeHox4*  *GpeHox5*  *GpeLox5*  *GpeHox7*  *GpeLox4*  *GpeLox2*  *GpePost1*  *GpePost2*  *GpeCdx*  *GpeGsx* | KX365100  KX365101  KX365102  KX365103  KX365104  KX365105  KX365106  KX365108  KX365107  KX365109  KX365110  KX365111  KX365112 |
| *Haliotis asinina* / Has | Mollusca | *HasHox3*  *HasHox4*  *HasHox5* | AAK17185  AAK11240  AAF78248 |
| *Homo sapiens* / Hsa | Chordata | *HsaGsx* | NP_573574 |
| *Idiosepius notoides* / Ino | Mollusca | *InoHox3*  *InoHox5*  *InoLox5*  *InoHox7*  *InoLox4*  *InoPost2*  *InoGsx* | KX365125  KX365126  KX365123  KX365124  KX365157  KX365127  KX365128 |
| *Lepisosteus oculatus* / Loc | Chordata | *LocGsx* | XP_006627824 |
| *Lingula anatina* / Lan | Brachiopoda | *LanHox1*  *LanHox3*  *LanHox5*  *LanLox5*  *LanHox7*  *LanLox4*  *LanLox2*  *LanPost1*  *LanPost2* | AAD45587  AAD45588  AAD45589  AAD45591  AAD45590  AAD45593  AAD45592  AAD45594  AAD45595 |
| *Lithobius atkinsoni* / Lat | Arthropoda | *LatHox1 LatHox2*  *LatHox3*  *LatHox4*  *LatHox5*  *LatHox7*  *LatHox8*  *LatAbdA*  *LatAbdB* | AAL36907  AAL36908  AAL36906  AAL36902  AAL36909  AAL36901  AAL36910  AAL36899  AAL36900 |
| *Lineus sanguineus* / Lsa | Nermertina | *LsaHox1*  *LsaHox3*  *LsaHox6* | CAA76295  CAA76296  CAA76297 |
| *Lottia* cf. *kogamogai* / Lko | Mollusca | *LkoHox1*  *LkoHox2*  *LkoHox3*  *LkoHox7*  *LkoLox4*  *LkoPost1*  *LkoPost2* | KX365129  KX365130  KX365158  KX365131  KX365132  KX365133  KX365134 |
| *Lottia gigantea* / Lgi | Mollusca | *LgiCdx* | XP_009065931 |
| *Mus musculus* / Mmu | Chordata | *MmuHox1*  *MmuHox2*  *MmuHox3*  *MmuHox4*  *MmuHox5*  *MmuHox6*  *MmuHox7* | NP_034579  NP_034581  NP_034582  NP_032291  NP_034583  NP_034584  NP_034585 |
| *Nymphon gracile* / Ngr | Arthropoda | *NgrHox1*  *NgrHox2*  *NgrHox5*  *NgrHox7* | ABD46723  ABD46725  ABD46729  ABD46732 |
| *Nereis virens** / Nvi | Annelida | *NviHox1 NviHox2*  *NviHox3*  *NviHox4*  *NviHox5*  *NviHox7*  *NviLox5*  *NviLox2*  *NviPost1*  *NviPost2*  *NviGsx* | AAD46166  AAD46167  AAD46168  AAD46169  AAD46170  ABD04657  AAD46174  AAD46171  AAD46175  AAD46176  ABB59695 |
| *Nucula tumidula* / Ntu | Mollusca | *NtuHox1*  *NtuHox2*  *NtuHox3*  *NtuHox4*  *NtuLox5*  *NtuLox4*  *NtuPost1*  *NtuPost2*  *NtuCdx*  *NtuXlox* | KX365135  KX365136  KX365137  KX365138  KX365139  KX365140  KX365141  KX365142  KX365143  KX365144 |
| *Patella vulgata* / Pvu | Mollusca | *PvuCdx* | CAD57266 |
| *Patiria miniata* / Pmi | Echinodermata | *PmiGsx* | AGK89736 |
| *Platynereis dumerilii* / Pdu | Annelida | *PduHox3*  *PduHox4*  *PduHox5*  *PduLox5*  *PduLox2*  *PduPost1*  *PduPost2*  *PduDlx1*  *PduEng*  *PduXlox*  *PduCdx* | ABD04656  ABD04658  ABD04660  ABD04654  ABD04659  ABD04653  ABD04651  CAJ38799  CAE46753  ACH87551  ACH87546 |
| *Priapulus caudatus* / Pca | Priapulida | *PcaGsx* | XP_014665932 |
| *Ptychodera flava* / Pfl | Hemichordata | *PflGsx* | AAR07642 |
| *Sacculina carcini* / Sca | Arthropoda | *ScaHox1*  *ScaHox2*  *ScaHox4*  *ScaHox5* | ABB46347  AAD00340  AAD00345  AAM50457 |
| *Saccoglossus kowalevskii* / Sko | Hemichordata | *SkoHox1*  *SkoHox2*  *SkoHox3*  *SkoHox4*  *SkoHox5*  *SkoHox6*  *SkoHox7* | AAP79296  ABK00018  AAP79286  AAP79297  ABK00019  ABK00020  AAP79287 |
| *Scutopus ventrolineatus* / Sve | Mollusca | *SveHox5*  *SveLox2*  *SveLox4* | KX365145  KX365146  KX365147 |
| *Strongylocentrotus purpuratus / Spu* | Echinodermata | *SpuXlox* | AAN17337 |
| *Symsagittifera roscoffensis* / Sro | Acoelomorpha | *SroHox1* | AAN11404 |
| *Tribolium castaneum* / Tca | Arthropoda | *TcaHox1*  *TcaHox3*  *TcaHox4*  *TcaHox5*  *TcaLox2* | AAK96034 AAK16424  AAK16423  AAK16422  AAL71874 |
| *Wirenia argentea*  / War | Mollusca | *WarHox3*  *WarHox4*  *WarHox5*  *WarLox5*  *WarHox7*  *WarLox4*  *WarLox2*  *WarPost1*  *WarPost2* | KX365148  KX365149  KX365150  KX365151  KX365152  KX365154  KX365153  KX365155  KX365156 |
| *Zootermopsis nevadensis* / Zne | Arthropoda | *ZneGsx* | KDR15663 |

*Current name *Alitta virens*
