# Supplementary material for: Comparative transcriptomics enlarges the toolkit of known developmental genes in mollusks
Source: BMC Genomics. 2016 Nov 10;17:905. doi: 10.1186/s12864-016-3080-9 (PMC5103448; doi:10.1186/s12864-016-3080-9)
Supplement: Additional file 2: Table S2. — Data used for the phylogenetic analysis of Hedgehog genes including the respective GenBank accession numbers. (DOC 31 kb) [file 12864_2016_3080_MOESM2_ESM.doc]

**Additional file 2: Table S2 Data used for the phylogenetic analysis of *Hedgehog* genes including the respective GenBank accession numbers.**

| **Superphylum** | **Species name** | **Accession Number** |
| --- | --- | --- |
| Deuterostomia | *Bos Taurus*  *Danio rerio*  *Gallus gallus*  *Pongo abelli*  *Xenopus tropicalis* | AF144100  AAC59742  AAA72428  XP_002818767  AAI66395 |
| Lophotrochozoa | *Acanthochitona crinita*  *Antalis entalis*  *Euprymna scolopes*  *Gymnomenia pellucida*  *Idiosepius notoides*  *Lottia* cf. *kogamogai*  *Lottia gigantea*  *Nucula tumidula*  *Octopus bimaculoides*  *Patella vulgata*  *Platynereis dumerilli*  *Perineis nuntia*  *Wirenia argentea* | KX365116  KX365118  AAV84106  KX365119  KX365120  KX365113  ACB42421  KX365121  AAZ99217  AAM60752  ADK38669  BAM74493  KX365122 |
| Ecdysozoa | *Aedes aegypti*  *Daphnia magna*  *Drosophila melanogaster Nasonia vitripennis* | EAT41674  BAJ05335  NP_001034065  XP_001605475 |
