# Supplementary material for: Comparative transcriptomics enlarges the toolkit of known developmental genes in mollusks
Source: BMC Genomics. 2016 Nov 10;17:905. doi: 10.1186/s12864-016-3080-9 (PMC5103448; doi:10.1186/s12864-016-3080-9)
Supplement: Additional file 3: Table S3. — Data used for the phylogenetic analysis of Hh-related genes including the respective GenBank accession numbers. (DOC 31 kb) [file 12864_2016_3080_MOESM3_ESM.doc]

**Additional file 3: Table S3 Data used for the phylogenetic analysis of *Hh-related* genes including the respective GenBank accession numbers.**

| **Phylum** | **Species name** | **Gene name** | **Accession Number** |
| --- | --- | --- | --- |
| Mollusca | *Lottia* cf. *kogamogai*  *Lottia* cf. *kogamogai*  *Lottia* cf. *kogamogai*  *Lottia* cf. *kogamogai*  *Lottia* cf. *kogamogai*  *Lottia* cf. *kogamogai*  *Lottia* cf. *kogamogai*  *Lottia* cf. *kogamogai*  *Lottia* cf. *kogamogai*  *Lottia* cf. *kogamogai*  *Lottia* cf. *kogamogai*  *Lottia gigantea*  *Lottia gigantea*  *Acanthochitona crinita*  *Acanthochitona crinita*  *Euprymna scolopes* | *Lophohog1*  *Lophohog2*  *HH-related1*  *HH-related2*  *HH-related3*  *HH-related4*  *HH-related5*  *HH-related6*  *HH-related7*  *HH-related8*  *HH-related9*  *Lophohog*  *HH-related*  *Lophohog*  *Hedgehog*  *Hedgehog* | KX365114  KX365115  KX395149  KX395150  KX395151  KX395152  KX395153  KX395154  KX395155  KX395156  KX395157  XP_009065705  XP_009066517 XP_009067386 XP_009049640 XP_009049641  KX365117  KX365116  AAV84106 |
| Annelida | *Capitella teleta* | *Lophohog* | ELU16390 |
